# Tri-Axial Dynamic Acceleration as a Proxy for Animal Energy Expenditure; Should We Be Summing Values or Calculating the Vector?

**DOI:** 10.1371/journal.pone.0031187

**Published:** 2012-02-17

**Authors:** Lama Qasem, Antonia Cardew, Alexis Wilson, Iwan Griffiths, Lewis G. Halsey, Emily L. C. Shepard, Adrian C. Gleiss, Rory Wilson

**Affiliations:** 1 Biological Sciences, College of Science, Swansea University, Swansea, Wales, United Kingdom; 2 Sports Science, College of Engineering, Swansea University, Swansea, Wales, United Kingdom; 3 Lon Gwynfryn, Swansea, United Kingdom; 4 School of Life Sciences, University of Roehampton, London, United Kingdom; 5 Centre for Fish and Fisheries Research, Murdoch University, Murdoch, Australia; Institut Pluridisciplinaire Hubert Curien, France

## Abstract

Dynamic body acceleration (*DBA*) has been used as a proxy for energy expenditure in logger-equipped animals, with researchers summing the acceleration (overall dynamic body acceleration - *ODBA*) from the three orthogonal axes of devices. The vector of the dynamic body acceleration (*VeDBA*) may be a better proxy so this study compared *ODBA* and *VeDBA* as proxies for rate of oxygen consumption using humans and 6 other species. Twenty-one humans on a treadmill ran at different speeds while equipped with two loggers, one in a straight orientation and the other skewed, while rate of oxygen consumption (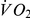) was recorded. Similar data were obtained from animals but using only one (straight) logger. In humans, both *ODBA* and *VeDBA* were good proxies for 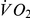 with all r^2^ values exceeding 0.88, although *ODBA* accounted for slightly but significantly more of the variation in 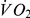 than did *VeDBA* (P<0.03). There were no significant differences between *ODBA* and *VeDBA* in terms of the change in 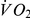 estimated by the acceleration data in a simulated situation of the logger being mounted straight but then becoming skewed (P = 0.744). In the animal study, *ODBA* and *VeDBA* were again good proxies for 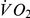 with all r^2^ values exceeding 0.70 although, again, *ODBA* accounted for slightly, but significantly, more of the variation in 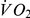 than did *VeDBA* (P<0.03). The simultaneous contraction of muscles, inserted variously for limb stability, may produce muscle oxygen use that at least partially equates with summing components to derive *DBA*. Thus, a vectorial summation to derive *DBA* cannot be assumed to be the more ‘correct’ calculation. However, although within the limitations of our simple study, *ODBA* appears a marginally better proxy for 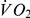. In the unusual situation where researchers are unable to guarantee at least reasonably consistent device orientation, they should use *VeDBA* as a proxy for 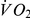.

## Introduction

The broad interest in animal optimal foraging [1] underpins the central concept that creatures should behave in such a way as to maximize their inclusive fitness by maximizing their net rate of energy intake [see 2,3]. This includes optimized harvesting solutions [e.g. 4], [5], [6] but also minimizing locomotion costs in animals that have to move to acquire food [7]. Thus, information on the rate at which organisms expend energy during movement is fundamental to informing models of optimal foraging and indeed, ultimately, the efficiency of movement affects the survival of wild animals [8]. Understanding optimality in foraging is only one example that demonstrates the importance of being able to determine energy expenditure but it, like many other biological processes, is best informed by energy expenditure at a fine-scale temporal resolution, something that is notably rare in published studies with some exceptions [9], [10], [11], [12]. This situation stems from a paucity of appropriate methods for determining the power use of wild animals.

The most common methods of measuring animal energy expenditure have used doubly labelled water (DLW) [13], [14], [15], [16], [17], direct and indirect calorimetry [e.g. 18], or some proxy for energy expenditure such as heart beat rate (fH) [19], [20], [21], [22], [23]. All the above systems have disadvantages [reviewed by 20], [24] which distill out into giving poor temporal resolution (doubly labelled water), being confined to a laboratory situation (calorimetry) or generally involving invasive methods of instrumentation (heart rate). See Halsey [25] and references therein for further details.

Beyond these approaches, however, some researchers have examined the use of mechanical motion sensors [26], [27] in studies of animal power use. In fact, as early as 1963, researchers proposed that the extent of body movement should act as a proxy for energy expenditure [28] because in order to elicit movement, animals need to expend energy, with more pronounced and vigorous movements presumed to arise as a result of more energy expended [e.g. 29], [30], [31]. Thus, specifically, energy expenditure should correlate with the extent of movement in some manner [32], [33]. In 2006, an acceleration-based proxy for energy expenditure focusing on dynamic body acceleration (*DBA*) was proposed, using tri-axial acceleration data derived from a logger recording at high rates (>10 Hz) and placed close to the participant's centre of gravity [34]. The specific proxy for metabolic rate was overall dynamic body acceleration (*ODBA*), determined by adding the dynamic acceleration from three orthogonally-placed accelerometers orientated so as to represent the main axes of the animal's body; in the surge, heave and sway dimensions [34]. Although this original work was conducted on birds, subsequent studies confirmed linear, strong correlations between the rate of oxygen consumption (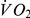) and *ODBA* in fish [35], amphibians [36], birds [34], [37], [38] and mammals [12], [39], including man [39].

Despite the promise of the newly proposed *ODBA* method, however, Gleiss, Wilson et. al. [24] point to an uncertainty in its formulation. Actually, acceleration is a vectorial quantity so it might seem incorrect that *ODBA* should treat each axis as independent because the implication is that the *ODBA* metric represents work done by three distinct straight-line paths and thus overestimates the work done for any specific movement. Furthermore, *ODBA* values are expected to differ according to the alignment of the axes of the logger with respect to the equipped participant, something that should not affect a vectorial solution [24]. The suggestion, therefore, is that properly calculated vectorial dynamic body acceleration (*VeDBA*) may, in fact, prove a better, and a more appropriate proxy for metabolic rate than does *ODBA*. Indeed, a recent study by McGregor, Busa et al. [40] uses *VeDBA* (although not referred to as such) rather than *ODBA* as a proxy of 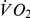. On the other hand, from a mathematical perspective both *ODBA* and *VeDBA* are norms and so are equally valid ways to measure the length of a vector. To ascertain which derivative is a better proxy of 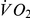 is difficult because there is no expected mathematical relationship that can be examined to calculate the impact of using different norms. Furthermore, the empirical relationship will depend on the species, the data logger location on the animal, and the behaviour and locomotion gait(s) of the animal. Thus a direct test of the predictive power of *ODBA* and *VeDBA* is required, yet Gleiss, Wilson et al. [24] note that no studies have explicitly sought to determine whether *ODBA* or *VeDBA* is a better predictor of metabolic rate and whether the outcome of such a test might be influenced by logger orientation on the animal.

The present study attempts to determine whether *ODBA* or *VeDBA* is a better proxy for rate of oxygen consumption. Humans are used as a model species [cf. 39] and primary data collected while people move at different speeds on a treadmill are analysed in detail. This is supplemented by reanalysis of published data [12] for six other animal species. The implications of the findings are discussed in terms of the most appropriate way to derive dynamic body acceleration in the future.

## Materials and Methods

### The human study

Twenty-one healthy adults (mean age ± SD: 20.44±3.28 years) were involved in the study. Before the start of the experiment, the height (1.75±0.07 m) and weight (70.66±9.78 kg) of the participants were measured according to the International Standards for Anthropometric Assessment (2001). The experimental protocol was approved by the Swansea University Ethics Committee. All participants were asked to give informed consent before the trials began.

Broadly, the investigations compared the rate of oxygen consumption during locomotion by humans on a treadmill while back-mounted loggers recorded tri-axial acceleration.

Specifically, all participants performed a VO_2_ max test [40] on a treadmill (Woodway Ergo ELG 55; Woodway GmbH, Germany) that started at 3 km/h and increased in speed every 3 min by 1 km/h until participant volitional exhaustion. During this process the participants breathed into a mask, and expired air was analyzed for oxygen and carbon dioxide content using an Oxycon Pro (Jaeger Oxycon Manual (Version 4.5), VIASYS Healthcare GmbH, Hoechberg, Germany) on a breath-by-breath basis. Acceleration was measured using two tri-axial accelerometers (X6-1A USB; Gulf Coast Data Concepts, LLC, Waveland, USA; 16 bit resolution, recording range ±6 g), each set to record at 80 Hz on each of the three orthogonal axes. The loggers were placed within holding moulds cut into a single polystyrene saddle to ensure correct orientation; one unit was mounted in accordance with the main body axes of surge, heave and sway while the other was set to be 30° displaced from this on all axes. This skew-mounted accelerometer was rotated by 30° about the roll, pitch and yaw axes respectively, where the roll axis was taken as the long axis of the accelerometer. The saddle was optimised by trial and error during pilot studies to move properly with the participant's body. It was placed in the centre of the participant's back between the shoulder blades and held in place using a specially made Silastic® (Silastic® P1 Base and Curing Agent, Thomson Bros Newcastle Ltd) harness which kept the system in a stable position even during the most vigorous of movement.

### The animal study

Data previously gathered comparing 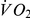
with acceleration data for animals during activity on a treadmill at Buenos Aires Zoo [12] were reanalyzed to supplement the work on humans. Species used were; coypu (*Myocastor coypus*) (4 individuals), larger hairy armadillo (*Chaetophractus villosus*) (1 individual), Muscovy duck (*Cairina moschata*) (1 individual), greylag goose (*Anser anser*) (2 individuals), Magellanic penguin (*Spheniscus magellanicus*) (2 individuals) and rockhopper penguin (*Eudyptes chrysocome*) (1 individual). Briefly, animals were equipped with acceleration data loggers, attached variously, before being exposed to a treadmill with the tread moving at a range of speeds between 0 and 2.52 km/h, the upper limit dependent on their capacities. The animals were given rests between the higher speeds where the predominant behavior was locomotion however at the lower speeds the animal typically exhibited a range of behaviors including searching, scratching and lying. An open circuitry respirometry system was used to measure 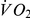. Full details of the protocol are given in Halsey et al. [12].

### Data analysis

The raw accelerometer data were converted to *DBA* by first smoothing each channel to derive the static acceleration using a running mean over 2 s [7] and then subtracting this static acceleration from the raw data [24]. The resulting values for dynamic acceleration were all then converted to positive values. These values for *DBA* were then either summed to provide *ODBA* [34];
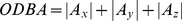
(1)where *A_x_*, *A_y_* and *A_z_* are the derived dynamic accelerations at any point in time corresponding to the three orthogonal axes of the accelerometer, or their vectorial sum (*VeDBA*) using;
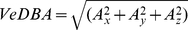
(2)Means for *ODBA* and *VeDBA* were derived for all data corresponding to particular running speeds (for each individual used in the experiments) and plotted against speed and 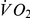. Because measurements of 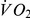 are most indicative of rate of energy expenditure when metabolism is mainly aerobic, 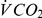 and 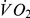 were also plotted against one another and the gas exchange threshold determined for each human participant using the v-slope method [41]: The plot of 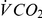 and 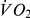 typically shows two slopes corresponding to the way 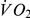 changes with respect to 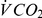 and the point at which these slopes intersect is considered to be the gas exchange threshold, which closely corresponds to the ventilatory threshold [41], [cf. 42], the point which approximately indicates when the participant changes from aerobic to anaerobic respiration as a main source of energy production. All data where participants were running at speeds which suggested that there was considerable anaerobic metabolism were excluded from the analysis.

Simple linear regressions were used to test the strength of relationships between *ODBA* and *VeDBA* for both humans and animals. Mixed linear models tested the relationships between data recorded from the straight mounting and data recorded from the skewed mounting in the human trials. The coefficient of variation for *ODBA* and *VeDBA* was calculated for each human participant for the two logger data sets combined. Mixed linear models were used to generate equations for 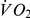 against the two acceleration metrics for all participants together, including participant as a random factor, separately for the straight- and skew-mounted logger data. To compare the error on estimates of 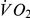 using *ODBA* or *VeDBA* caused by an acceleration logger becoming skewed, the difference between measured 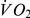 and 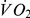 estimated by a skew-mounted logger using calibrations for a straight-mounted logger at speed 5 (an average walking speed) was calculated for both these derived metrics. Paired tests were used to test for differences between *ODBA* and *VeDBA*. Mean values are provided ±1 S.E. (standard error).

## Results

### The human study

**Figure 1:**
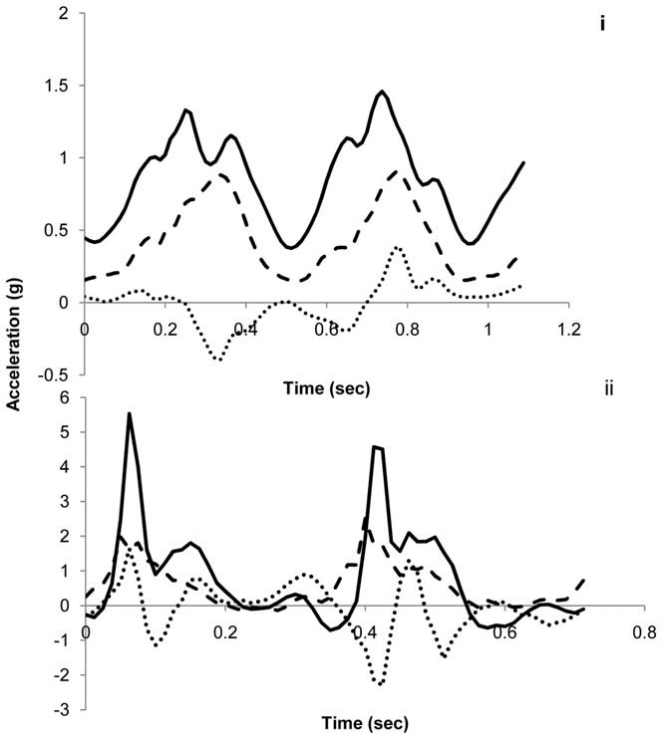
Heave (continuous line), sway (dotted line) and surge (dashed line) acceleration axes displayed graphically over one stride (from each leg) during walking (i) and running (ii).

The accelerometers recorded a very precise profile of tri-axial acceleration from each participant during walking and running (Fig. 1) with clear peaks in heave and surge in particular, denoting each stride, although peaks in sway were also apparent.

Gas analytical data and data from the straight-mounted logger were obtained for all participants, while data from the skew-mounted logger were obtained for 18 of the participants (on three occasions the logger failed). Q-Q plots indicated that the distribution for all the 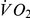
s data together was reasonably normal. *ODBA* and *VeDBA* appeared non-normally distributed as did the values representing the percentage change in estimated 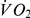 due to the logger becoming skewed. However, for the majority of analyses, *ODBA* and *VeDBA* represented the independent variable. The r^2^ values for 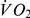 against *ODBA* and *VeDBA* for both straight and skewed logger orientations were reasonably normal.

**Figure 2:**
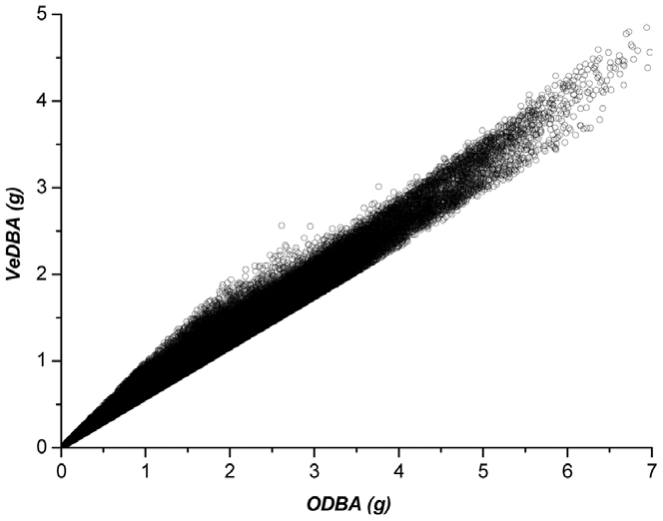
Instantaneous *ODBA* plotted against *VeDBA* using all data from a participant recorded during a full 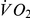 max test. In this example, as with all other participants, the relationship between *ODBA* and *VeDBA* was highly significant (*VeDBA* = 0.014+0.6418 *ODBA*, r^2^ = 0.987, P<0.001).

**Figure 3:**
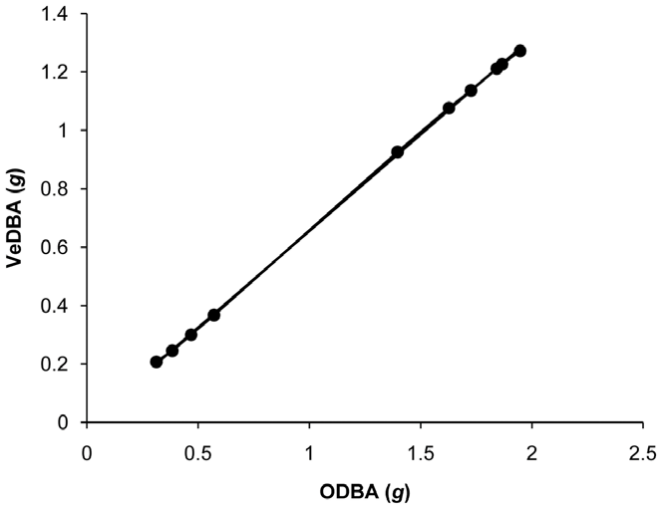
Relationship between mean *ODBA* and mean *VeDBA* (means taken for each running speed) for a test participant during a 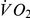 max test. Only data during the period when the participant did not exceed the ventilatory threshold (for definition see text) are included. as with all other participants, the relationship between *ODBA* and *VeDBA* was highly significant (*VeDBA* = 0.014+0.6418 *ODBA*, r^2^ = 0.987, P<0.001).

**Figure 4:**
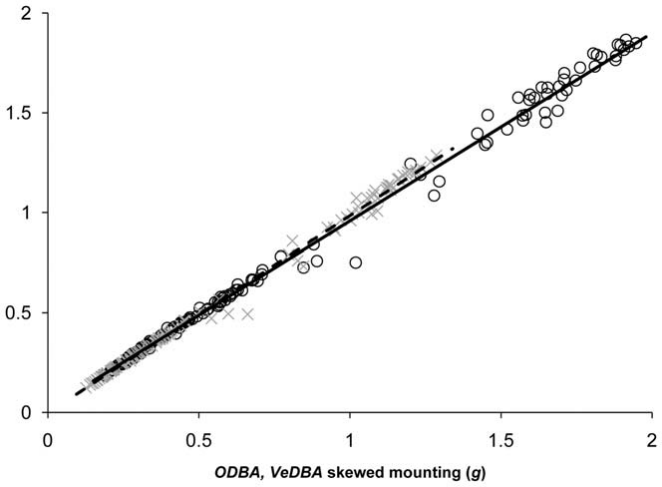
Dynamic body accelerations (*ODBA* – circles, and *VeDBA* –crosses) from straight- versus skew-mounted accelerometers (for details see text). Each point denotes a mean value derived from a three-minute trial of a participant moving at one particular speed below the lactate threshold. Data from all participants are included.

**Figure 5:**
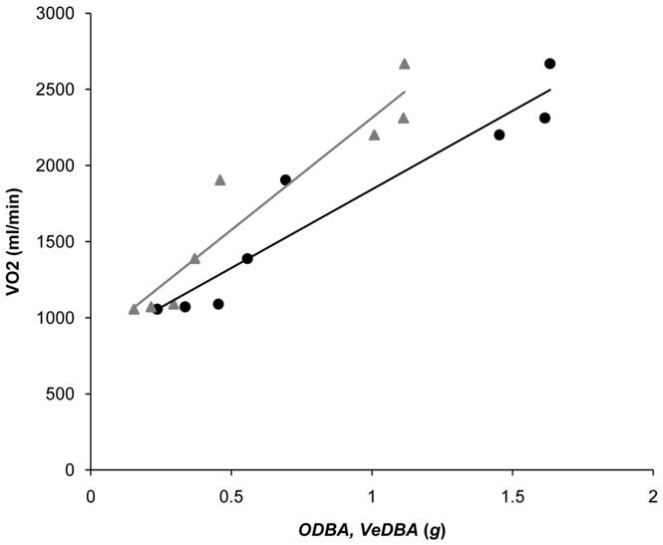
An example plot of 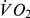 uptake against *ODBA* (black circles) and *VeDBA* (grey triangles) over the duration of the trial following removal of the points above the participant's anaerobic threshold.

For all participants and both logger mountings, *ODBA* and *VeDBA* from trials were highly correlated with each other (Figs. 2 and 3), with r^2^ values on means derived from all participants typically being *ca*. 0.999. Mixed linear models (straight *ODBA*∼skewed *ODBA*+participant(random), or, straight *VeDBA*∼skewed *VeDBA*+participant(random)) indicated that during the trials, *ODBA* values from the straight-mounted devices were highly correlated with the *ODBA* values from the skew-mounted devices (r^2^ = 0.99), as were *VeDBA* values from the straight- and skew-mounted devices (r^2^ = 0.99) (Fig. 4). Both *ODBA* and *VeDBA* were highly correlated with 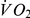 (Fig. 5). Mixed linear models (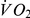
∼*ODBA*+participant(random), or, 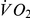
∼*VeDBA*+participant(random)) returned significant relationships for both the straight and the skewed mountings (Table 1).

**Table 1:** Overall relationships between 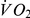 and *ODBA* or *VeDBA* recorded for humans locomoting on a treadmill using an acceleration logger in a straight orientation or a skewed orientation.

	Straight	Skewed
*ODBA*	*VO_2_* = 1132.*ODBA*+615 r^2^ = 0.915	*VO_2_* = 1466.*ODBA*+776 r^2^ = 0.94
*VeDBA*	*VO_2_* = 1664.*VeDBA*+636 r^2^ = 0.914	*VO_2_* = 1659.*VeDBA*+629 r^2^ = 0.91

*ODBA* accounted for significantly more of the variation in 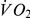 than did *VeDBA* for the straight-mounted loggers (mean r^2^, *ODBA*: 0.95±0.01; *VeDBA*: 0.94±0.01; t_20_ = 2.29, P = 0.03) and for the skew-mounted logger (t_17_ = 2.44, p = 0.03) (mean r^2^, *ODBA*: 0.94±0.01; *VeDBA*: 0.91±0.01). The difference in r^2^ values for single linear regressions of 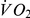 against *ODBA* versus 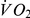 against *VeDBA* for each participant, for both the straight-mounted logger and the skew-mounted logger, are very small. 95% confidence intervals derived from paired t tests indicate that the true difference in r^2^ for the straight-mounted logger is in the range of 0.0004 to 0.0078, while for the skew-mounted logger is in the range of 0.0003 to 0.0040. These ranges represent less than 0.1% of mean r^2^ values.

To test for differences between *ODBA* and *VeDBA* in the effect on estimates of 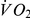 in the case of an initially straight-mounted logger subsequently becoming skewed 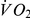 measured during speed 5 on the treadmill was compared to 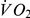 estimated from acceleration measurements recorded by the skew-mounted logger using the straight-mounted logger calibrations. For both *ODBA* and *VeDBA* the percentage difference between 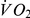 measured and 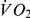 estimated was small (median, *ODBA*: 0.93; *VeDBA*: 0.81), and the size of this difference was similar for the two metrics (Wilcoxon signed ranks test: Z = −0.327, N = 18, P = 0.744).

#### Table 1

Overall relationships between 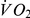 and *ODBA* or *VeDBA* recorded for humans locomoting on a treadmill using an acceleration logger in a straight orientation or a skewed orientation.

### The animal study

In a manner similar to the human study, the coefficients of determination for the relationships between *ODBA* or *VeDBA* and 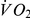 were all high, ranging between r^2^ = 0.70 for the *VeDBA versus* 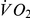 relationship for coypu 4 and r^2^ = 0.99 for the *VeDBA versus* 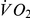 relationship for the rockhopper penguin (Table 2). The *ODBA* values had significantly higher coefficients of determination than the *VeDBA* values (t = 2.54, P<0.03).

**Table 2:** r^2^-values for relationships between *ODBA* and *VeDBA* and 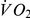 recorded using an acceleration data logger on a range of animals during activity at Buenos Aires Zoo.

Species	*ODBA*	*VeDBA*
*Chaetophractus villosus*	0.9775	0.942
*Myocastor coypus 1*	0.9594	0.94
*Myocastor coypus 2*	0.7449	0.7019
*Myocastor coypus 3*	0.9473	0.9486
*Myocastor coypus 4*	0.8617	0.8568
*Cairina moschata*	0.9853	0.9841
*Anser anser 1*	0.9022	0.8904
*Anser anser 2*	0.9427	0.9242
*Spheniscus magellanicus 1*	0.975	0.9662
*Spheniscus magellanicus 2*	0.8979	0.811
*Eudyptes chrysocome*	0.9914	0.9957

#### Table 2

r^2^-values for relationships between *ODBA* or *VeDBA* and 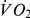 recorded using an acceleration data logger on a range of animals during activity at Buenos Aires Zoo.

## Discussion

In the purely physical sense, *ODBA* and *VeDBA* are derived using the same terms and, although the relative importance of the terms differs, the precise formulation of them means that larger *VeDBA* values will generally also accompany larger *ODBA* values, although *VeDBA* values will almost invariably be lower and never higher than *ODBA*. How much higher *ODBA* is than *VeDBA* will depend, *inter alia*, on the type of motion recorded which, in turn depends on animal type, gait and tag location. Our study was limited in scope, incorporating data from only 7 species, all of which were travelling in a straight line on a treadmill (although some species exhibited a range of behaviors at slower speeds), so it is unwise to over-interpret. Nonetheless, the treadmill approach has been used as a general method to simulate increased activity of all types by researchers examining the relationship between heart rate and 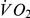 for many years [e.g. 20], [21] and two studies have explicitly sought to incorporate behaviours other than straight-line treadmill locomotion within the treadmill context with success [38], [43]. With these provisos in mind, generally, it is to be expected that an important finding of this study is the close correlation between *ODBA* and *VeDBA* where no human participant had an r^2^ of less than 0.998 for mean values derived from either skew or straight loggers with, unsurprisingly, the slope of the relationship always being less than 1. In addition, simple comparisons of the correlations between *ODBA* and *VeDBA* with 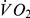 in animals show that they differ minimally (Table 2). This makes the discussion of whether *ODBA* or *VeDBA* is a better predictor of metabolic rate [24] rather academic. Nonetheless, given concerns about potential differences in the utility of *ODBA* with respect to *VeDBA* [24], the present study was conducted to carry out tests into the matter.

### Vectorial versus summed tri-axial acceleration as a proxy for 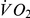


**Figure 6:**
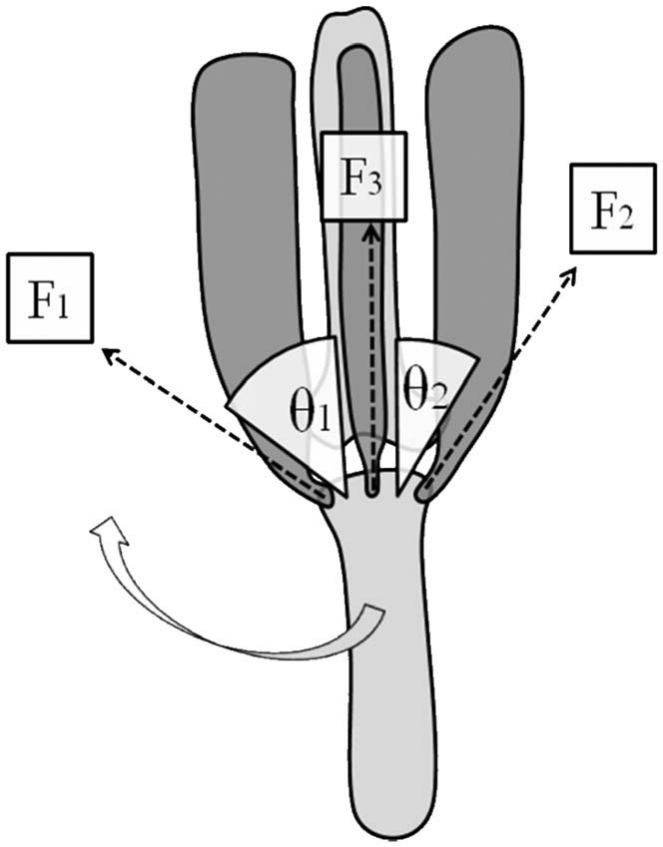
Schematic representation of a movement arc (curved arrow) elicited by one bone (light grey) with respect to another and brought about by contraction of multiple muscles (dark grey) with varying forces (*F*) with differing angles of insertion (*θ*).

From a theoretical standpoint it may seem perplexing that *VeDBA* does not outperform *ODBA* as a proxy for 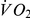 and a specific explanation is warranted. If the simple scenario of one limb articulating on another is considered (Fig. 6) where muscles emanating from the upper limb are inserted at various angles (*θ*) on the lower limb [e.g. 44], each exerting a force (*F*), then the overall force along the longitudinal y-axis (*Fy_tot_*) is given by the vectorial solution;
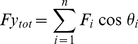
(3)where the subscripts refer to each of the specific muscles with their defined forces and angles of insertion relative to the *y*-axis of the lower limb. In a similar manner, the total force along axis *x* is;
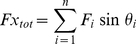
(4) The torque (*τ*) along the y-axis produced by the contraction of these muscles depends on the overall force generated along that axis by each muscle (Eq 3) and the moment arm (*d*), defined as the perpendicular distance between the line of action of the muscle force and the pivot point of the articulation so that;
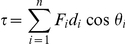
 The torque is related to the angular acceleration (*α*) via;
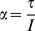
(6)where *I* is the moment of inertia. The linear acceleration (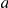) perceived by an accelerometer placed on the moving lower limb is dependent on the distance between pivot and transducer (*r*) by;
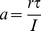
(7) Thus, the (linear) acceleration perceived by an accelerometer mounted in the y-axis and measuring in the plane of movement can be determined by substituting Eq (5) into equation (7) and is approximated by;
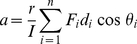
(8)which is clearly a vectorial solution. However, the work done (*W*) during muscular contraction to produce the forces necessary for the movement is given by;
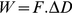
(9)for each muscle involved, where *ΔD* is the distance contracted. The total amount of energy used during contraction by all the muscles involved in moving the limb (*W_to_*_t_) is;
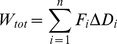
(10)a necessarily non-vectorial derivation, where the energy used equates directly with the oxygen consumed [45]. Thus, seen from a pure physics perspective, *VeDBA* is the proper way to derive the total magnitude of the acceleration vector at any one moment in time. However, the issue of interest to biologists studying energetics is not the total acceleration but how the *DBA* signal relates to rate of energy expenditure, and specifically the rate of energy expenditure used by the muscles involved. The rate of energy expenditure is not just dependent on the movement arc, which is described by *VeDBA* [see 24 for treatment of this], but also dependent, among other things, on the force exerted by the contracting muscles at the points of their insertion. A single muscle can contract to produce a movement arc of one limb by exerting an appropriate force (Fig. 6) while exactly the same movement arc can result from the contraction of two or more differently inserted muscles (Fig. 6), each of which exerts a force that leads to a vectorial solution that accords with that exhibited by the single muscle. In both cases the overall result for movement and physical work done is the same but in the latter case the oxygen consumed by the multiple muscles will exceed that of the single muscle because forces are developed that are not equally manifest in the movement. Fundamental to the amount of oxygen used by a body is the amount of muscle tissue that is active [46] and the precise orientation of various muscle groups involved in limb movement is critical in this regard. Human walking and running is brought about by a complex interplay of interacting, and variously inserted, muscles [44] which, nonetheless, produces a relatively simple movement arc which equates to the vectorial component of the variously contracting muscles even though the muscular work produced may more appropriately be represented by a sum value of muscular contraction. The ‘inefficiencies’ that result from partially opposing contracting muscles are, in fact, necessary for increasing limb stability [cf. 8]. For example, animals moving over rough terrain could not afford to have limb movement that is overly sensitive to lateral forces.

### Skew versus straight-mounted logger orientation

A specific concern, and one that perhaps might lead to the greatest discrepancy between *ODBA* and *VeDBA*, is what happens when device orientation is not standardized. Importantly, in our study on humans, the difference in recorded 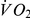 at a speed of 5 km/h compared to 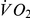 estimated for the same speed from the data recorded by the skew-mounted logger using the calibrations obtained from the straight-mounted logger was small. Further, it did not differ between *ODBA* and *VeDBA*. Thus, apparently, even if a logger is deployed in the straight position but then subsequently slips out of true, perhaps, for example, due to the intensity of the exercise, both *ODBA* and *VeDBA* would appear similarly powerful proxies for 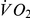. In fact, contrary to what might be expected from a purely physical treatise, *VeDBA* did not outperform *ODBA* based on any of statistical analyses conducted.

The actual acceleration values recorded by a straight- with respect to skew-mounted tri-axial accelerometer can be derived for any scenario by considering the relative rotations for each of the axes. Here, the matrix representation for the acceleration vector transformation is
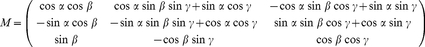
(11)where 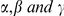 are the angles of roll, pitch and yaw angles of the skew-mounted accelerometer relative to the straight-mounted one, where the rotations are carried out in that order.

Thus, if the acceleration vector measured by the device in the straight-mounted position is
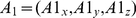
(12)then in the skew-mounted position, that same acceleration is measured as a vector;
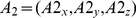
(13)where
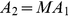
(14)


**Figure 7:**
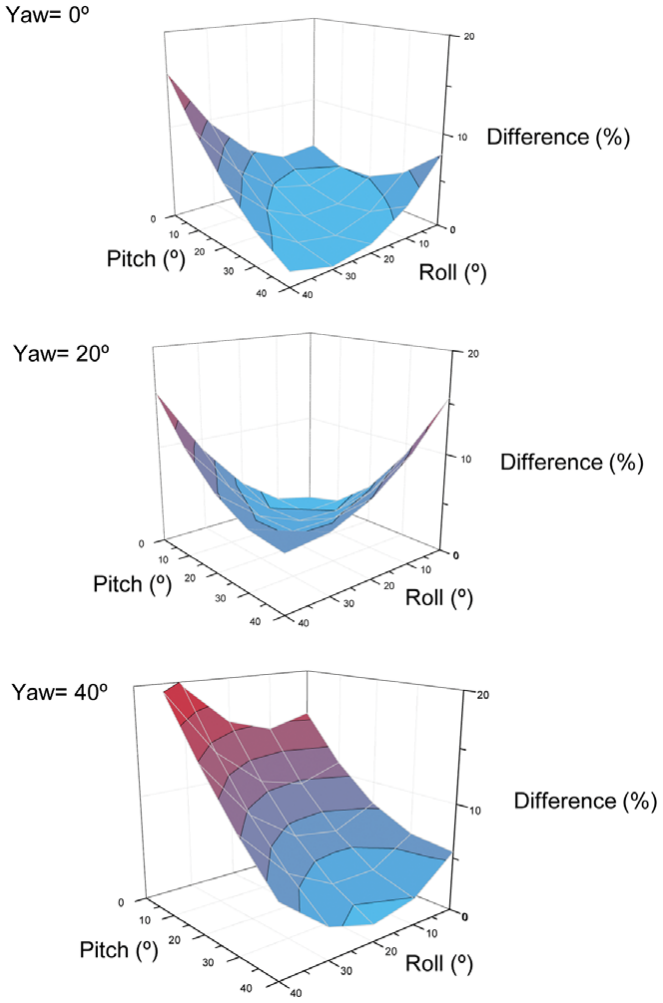
Predicted difference between straight- and skew-mounted *ODBA* derived from recordings on a tri-axial accelerometer subjected to equal acceleration in the heave, surge and sway axes as a function of pitch, roll and yaw differences between straight and skew. Contour lines show 2.5% intervals.

Derived values for these vector components can be used to produce *VeDBA*, (using Eq 2) which does not change with orientation and, more particularly, to produce *ODBA* (using Eq 1) which does change with orientation (Fig. 7). This approach shows that under given conditions of triaxial acceleration (the case shown in Fig. 7 shows equal amounts of dynamic acceleration in the heave surge and sway axes), deviations of up to 10° in any one axis produce only up to a 1% change in *ODBA* (although a 10% change in two or three axes simultaneously produces about a 1% and 0.03% change in *ODBA*, respectively). In fact, to produce a 5% change in *ODBA* requires skew placement of >20° in one or more axes, something which would be immediately obvious during many device attachment protocols. Thus, although many situations where loggers containing tri-axial accelerometers are attached to animals for derivation of energy expenditure will not have to worry overly about orientation [e.g. 47], there are a number of obvious situations, such as having round, unmarked devices in which the orientation of the transducers is unclear and placing devices via suction cups onto whales [48], [49] that should only use *VeDBA*. Importantly though, the latter situation is also likely to incur substantial additional errors in any derivation of *DBA* and its relation to 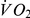 resulting from the non-standardized positioning of the device on the body relative to the animal's centre of gravity, which would markedly affect both *ODBA* and *VeDBA* signals [24]. This effect is apparent even in our results on humans where, despite placing the straight and skew tag as closely together as we could, the correlation coefficient between 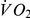 and *VeDBA* for straight and skewed tag orientations was (marginally) different. Allusion to this phenomenon, in an albeit simplistic form, can be accessed via equation (7) which shows the extent to which distance from the pivot point (or distance from the moving body part such as a fish's tail [24]) affects *DBA*.

Overall, our results showed that *ODBA* was insensitive to the accelerometer when its axes were skewed off the major axes of the body (by 30° in roll, pitch and yaw), which we attribute to the general variance between 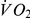 and *DBA* and the fact that the degree of skew tested was insufficient to elicit a marked difference in the way *ODBA* reacts to changes in orientation. Thus, against predictions based on physical theory, our study indicates that *ODBA* is, in fact, not worse than *VeDBA* at predicting 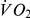; if anything it is better (though the difference is minimal) as long as devices can be attached close to the major axes of the body. This indicates that whether researchers use *ODBA* or *VeDBA* may not be the most critical issue in treatment of *DBA* signals and 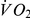 because variation in device positioning is likely to introduce much more variability [39]. Future work will have to address this aspect more carefully. For the moment, researchers should certainly be working towards positioning their devices in the same anatomical location as far as possible and, with the exception of a few species such as cetaceans [47] and animals that have tags implanted [50], this will tend to lead to device orientation being correct anyway. There are other particularly germane reasons for researchers to orientate devices on their study species in a comparable manner. In particular, it underpins powerful behaviour identification protocols based on posture and dynamic acceleration [51], a process which, itself, requires device orientation to be controlled rigorously. Subsequently, both the known behaviour and the *ODBA-*derived estimate of metabolic rate can be used to determine activity-specific metabolic rate. *VeDBA* would, however, clearly be a better proxy of 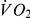 than *ODBA* where device orientation cannot be maintained within a 30° arc in any of the angular dimensions and so should be used when loggers cannot be implanted [52] reasonably precisely, or attached reasonably precisely [50], or are ingested [e.g. 53]. Importantly though, determination of behaviour using inconsistently placed accelerometers is more problematic.

### Conclusions

The assumption that *DBA* derived by a vectorial rather than an absolute summation is more appropriate as a proxy for 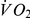 is not founded for devices mounted in a standardized manner and issues of force generation by muscles likely account for this rather than just the physics associated with measures of acceleration. *ODBA* and *VeDBA* are very closely correlated with each other and both can be excellent proxies for movement-based metabolic rate. Proponents of DBA as a proxy of metabolic rate must choose which derivation to use based on (a) the value they place on the derivation representing the biology of muscle metabolism (b) whether they are concerned that logger orientation could vary markedly (c) whether they wish to compare their DBA values with values in the literature. Critically, neither *ODBA* nor *VeDBA* deals with the problem of variation in device positioning.
